# Paraneoplastic Lambert–Eaton myasthenic syndrome associated with non-small cell lung cancer: data from the European LEMS registry and systematic review

**DOI:** 10.1186/s42466-025-00453-5

**Published:** 2025-12-08

**Authors:** Hannah Preßler, Imène Haddy, Claire Daugherty, Ville Postila, Andreas Meisel

**Affiliations:** 1 Department of Neurology With Experimental Neurology, Charité-Universitätsmedizin Berlin, Charitéplatz 1, 10117 Berlin, Germany; 2 German Center for Neurodegenerative Diseases (DZNE), Berlin, Berlin, Germany; 3 Charité-Universitätsmedizin Berlin, Neuroscience Clinical Research Center Berlin, Berlin, Germany; 4 SERB Pharmaceutical West Conshohocken, West Conshohocken, PA USA; 5 SERB SA, Brussels, Belgium; 6 Center for Stroke Research Berlin, Charité-Universitätsmedizin Berlin, Berlin, Germany; 7 Integrated Myasthenia Gravis Center, Charité-Universitätsmedizin Berlin, Berlin, Germany

**Keywords:** Lambert-Eaton myasthenic syndrome, Paraneoplastic, Tumor, Cancer treatment, Ataxia

## Abstract

**Background:**

Paraneoplastic Lambert-Eaton myasthenic syndrome (pLEMS) is well-established in small-cell lung cancer (SCLC), but data on other malignancies are limited. We aimed to define the clinical phenotype of pLEMS in non-SCLC cancers (non-SCLC-pLEMS) relative to SCLC-associated LEMS (SCLC-pLEMS) and autoimmune LEMS (aiLEMS).

**Methods:**

Retrospective analysis was conducted to compare patients with SCLC-pLEMS, aiLEMS and non-SCLC-pLEMS from the European LEMS registry, and further non-SCLC-pLEMS cases were identified by a systematic review following the Preferred Reporting Items for Systematic Reviews and Meta-Analyses (PRISMA) guidelines.

**Results:**

The registry included 72 aiLEMS, 12 SCLC-pLEMS, and 11 non-SCLC-pLEMS patients. LEMS preceded cancer diagnosis in 33% of SCLC-pLEMS (median 2 months) and 30% of non-SCLC-pLEMS (median 15 months), was concurrent in 25% and 30% and followed tumor diagnosis in the remainder. At study enrollement, Quantitative Myasthenia Gravis (QMG) scores were higher in SCLC-pLEMS (median 12, range 1–24) with recent tumor therapy initiation (median 3 months), and lower in non-SCLC-pLEMS (median 6, range 0–19), with longer (median 12 months) or completed tumor therapy, and aiLEMS (median 5, range 0–23). During follow-up, QMG improved with tumor therapy, and worsened with recurrence/progression in pLEMS groups. After completion of cancer treatment, QMG values in SCLC-pLEMS (median 6, range 0–19) and non-SCLC-pLEMS (median 5, range 1–22) were comparable to each other and to aiLEMS (median 7, range 0–29). Ataxia was significantly more frequent in SCLC-pLEMS (64%) and non-SCLC-pLEMS (55%) than in aiLEMS (19%, *p* = 0.006 and *p *= 0.024). Another 115 literature-reported non-SCLC-pLEMS cases were identified (total n = 126, comprising 137 tumors). Most common were non-small cell lung cancer (NSCLC) (n = 25, 18%), Merkel cell carcinoma (n = 18, 13%) and lymphoproliferative disorders (n = 15, 11%). In 52 literature-reported LEMS patients with outcome data, 88% partially or fully recovered after tumor therapy, leaving the paraneoplastic origin uncertain in many.

**Conclusions:**

Our results suggest that, beyond SCLC, other tumors can trigger pLEMS. Compared with aiLEMS, non-SCLC-pLEMS and SCLC-pLEMS showed a higher frequency of ataxia, and LEMS severity tended to reflect tumor treatment status, while disease severity becomes comparable across subtypes after cancer therapy. The frequent improvement of symptoms with tumor-directed treatment supports extended screening beyond SCLC and timely management.

**Supplementary Information:**

The online version contains supplementary material available at 10.1186/s42466-025-00453-5.

## Background

Lambert-Eaton myasthenic syndrome (LEMS) is a very rare neuromuscular autoimmune disease, characterized by proximal muscle weakness, increased fatigability, reduced tendon reflexes, and autonomic dysfunction. Antibodies (Abs) that target presynaptic voltage-gated calcium channels (VGCC) are found in approximately 90% of patients [1]. Up to 60% of LEMS occur as a paraneoplastic disorder (pLEMS), most commonly small cell lung cancer (SCLC-pLEMS), a smoking-related neuroendocrine tumor, that express functional VGCC [2–4]. Over the past decades, LEMS has been recognized in various non-SCLC malignancies (non-SCLC-pLEMS), including non-small-cell lung and mixed carcinomas [5–9], thymoma [10–12], and prostate cancer [13–17]. In a small cohort of patients with prostate cancer exhibiting neuroendocrine and small-cell characteristics, LEMS symptoms correlated with symptom activity [18]. Similarly, Merkel cell carcinoma (MCC), a neuroendocrine tumor, has been identified in a broader range of paraneoplastic neurologic syndromes (PNSs), with LEMS being among the most frequent [19]. However, the link between LEMS and certain tumor types, such as lymphoproliferative disorders, remains unclear [20–25].

Notably, in a recent Turkish study, only just over the half of pLEMS cases were associated with SCLC [26]. Given the importance of early detection and treatment of all tumor types potentially associated with LEMS, a better understanding of LEMS phenotypes beyond SCLC is necessary. So far, it remains unclear whether non-SCLC-pLEMS represents a paraneoplastic manifestation or a coincidental finding. Meanwhile, autoimmune LEMS (aiLEMS) corresponding to non-tumour or idiopathic LEMS—is considered the non-paraneoplastic form of the disease. Therefore, we aimed to characterize non-SCLC-pLEMS in detail and compare it with SCLC-pLEMS and aiLEMS. In addition, we conducted a systematic literature review to identify reported non-SCLC-pLEMS cases and compared them with registry-based non-SCLC-pLEMS to assess the representativeness of our registry cohort.

## Methods

### Patient selection from the European LEMS registry

In this study, we used data from the European LEMS registry (LEMS-01, EUPAS6106, Study ID 39278), including 30 centers in Germany, Italy, France, Spain, and United Kingdom, to retrospectively categorize LEMS cases recorded between May 2010 and August 2019. LEMS diagnosis was based on clinical evaluation, abnormal neurophysiological testing, and/or a positive VGCC antibody result [27, 28]. LEMS cases with non-SCLC tumors were assigned to the non-SCLC-pLEMS group, and patients with multiple tumors including SCLC to the SCLC-pLEMS group. Classification was further based on clinically relevant timeframe between tumor diagnosis and LEMS onset and by the response of LEMS symptoms to tumor therapy. Patients underwent standard assessments including Quantitative Myasthenia Gravis (QMG) score, muscle strength, reflexes, ataxia, autonomic nervous system (ANS) function and activities of daily functioning. Ataxia was assessed through several tests (line walking, Romberg’s test, finger-to-nose test and heel-to knee-test) and considered present if at least two of these tests were positive. Data on demographics, smoking, other paraneoplastic neurologic syndrome (PNS), and treatments. LEMS specific treatment included symptomatic therapies (amifampridine, amifampridine phosphate, pyridostigmine, aminopyridine), steroids, non-steroidal immunosuppressants (azathioprine, methotrexate, mycophenolate mofetil, cyclosporine), cyclophosphamide, rituximab, exacerbation therapies (intravenous immunoglobulins, plasma exchange, immunoadsorption), and tumor therapies. Clinical evaluations were conducted at enrollment and annually or biannually.

### Systematic review

**Fig. 1:**
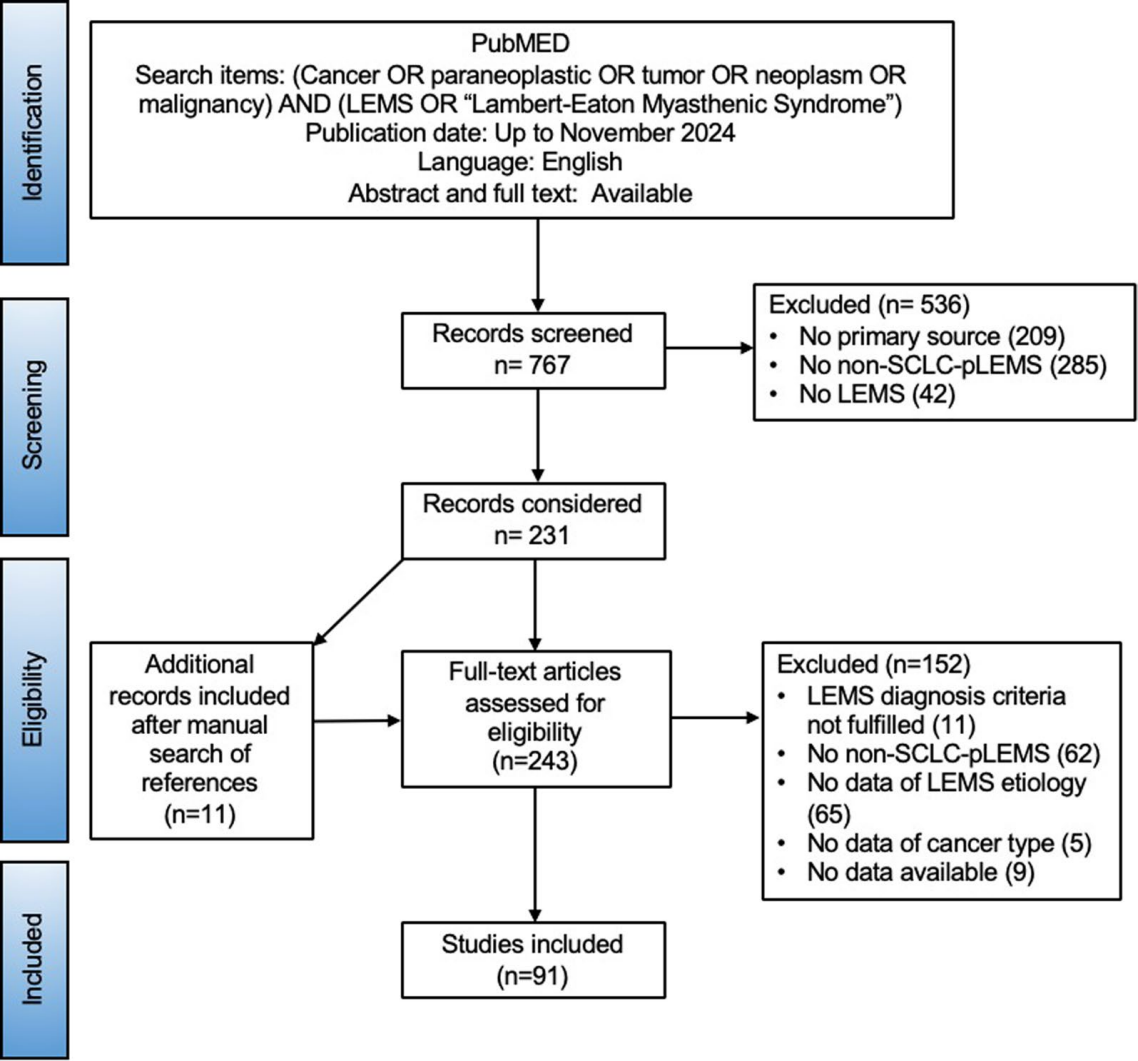
PRISMA flow chart illustrating number of studies screened, included, and excluded

The review was conducted in accordance with the guidelines of the Preferred Reporting Items for Systematic Reviews and Meta-Analyses (PRISMA) (Fig. 1). A systematic search of articles published from 1979 to 2024, was performed on MEDLINE/PubMed using the following MeSH search terms, free text, and related search terms: (Cancer OR paraneoplastic OR tumor OR neoplasm OR malignancy) AND (LEMS OR “Lambert-Eaton Myasthenic Syndrome”). Additional relevant publications in the literature search were identified by examining reference lists of the selected articles. Articles and abstracts were screened and only studies with full text available were further included. Screening was initially performed by one reviewer (IH); in cases of ambiguity, a second reviewer (HP) was consulted. When multiple publications reported on the same patient cohort, duplicate data were excluded, prioritizing the larger or more comprehensive dataset when necessary. We focused our literature review on non-SCLC-pLEMS cases to evaluate the representativeness of our registry data, as large studies have already provided comprehensive clinical data on SCLC-pLEMS [18, 29]. All reported non-SCLC cancer types in patients with LEMS were summarized. A subset of cases with available patient-level data, in which the LEMS diagnosis was based on the same diagnostic criteria applied in the registry, was analyzed for demographics, clinical phenotype, time relationship between tumor diagnosis and LEMS onset, treatment approaches, and outcomes in relation to tumor therapy.

### Statistical analysis

First, registry patient characteristics were analyzed, including comparisons among LEMS groups. Second, literature reported non-SCLC-pLEMS cases were identified via a PRISMA-guided search, with patient-level data extracted when available and comparisons made between neuroendocrine and non-neuroendocrine cancers. Registry-based, and literature-based non-SCLC-pLEMS were compared for clinical characteristics. Data were summarized as medians and ranges or frequencies and percentages, as appropriate. Categorical variables were compared with Chi Square test or Fisher’s exact test. For continuous variables, which were not normally distributed, the Mann Whitney U test and Kruskal Wallis test were used. Overall survival (time from LEMS onset to death) was estimated with Kaplan–Meier curves; patients alive were censored at last follow-up. Groups were compared with two-sided log-rank tests (*α* = 0.05), and median survival with 95% CIs was derived from the Kaplan–Meier estimates. Due to an exploratory study design, no adjustments were made for multiple testing, and a significance level alpha of 0.05 was chosen. Analyses were solely based on the available information with no imputation for missing data. Data analysis and figure creation were performed in Python (version 6.5.7) using the indicated Python packages (matplotlib, numby, pandas).

## Results

### European LEMS registry cohort

Of 105 patients enrolled in the European LEMS registry between 2010 and 2019, 96 were eligible. One patient with lung cancer was excluded due to missing histology, leaving 95 patients for analysis. Among them, 23 of 95 patients (24%) were diagnosed with pLEMS and 72 of 95 (76%) with aiLEMS. Within the pLEMS group, 12 of 23 patients (52%) had SCLC-pLEMS while 11 patients (48%) had non-SCLC-pLEMS. The non-SCLC-pLEMS group comprised 11 patients with 13 tumors, including MCC (n = 2), lymphoproliferative disorders (n = 2), thymoma (n = 2), breast cancer (n = 1), colon cancer (n = 2), pancreatic cancer (n = 1), laryngeal cancer (n = 1), esophageal cancer (n = 1), and rectal cancer (n = 1), three of these were neuroendocrine (MCC and pancreatic cancer).

**Fig. 2:**
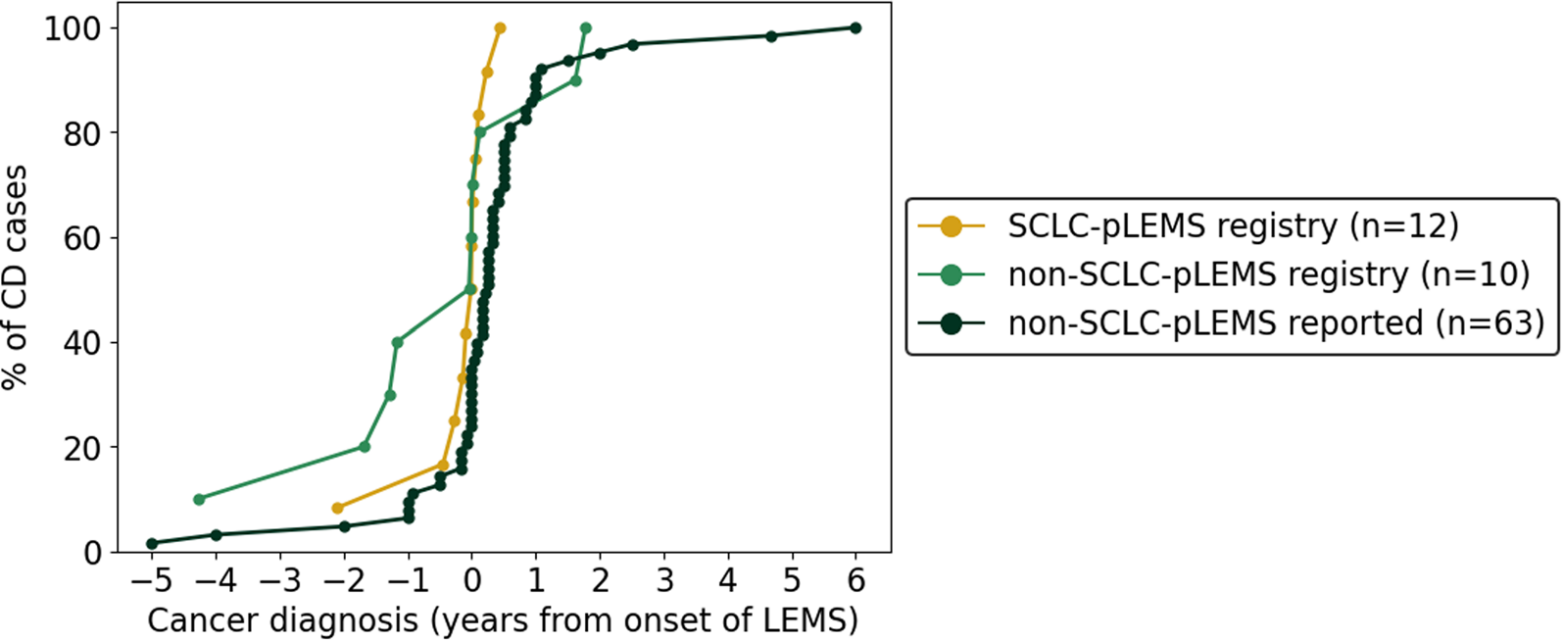
Time relationship of cancer diagnosis with the onset of LEMS in SCLC-pLEMS and non-SCLC-pLEMS (registry and literature). For one non-SCLC-pLEMS patient with two malignancies, the first tumor’s diagnosis date was used. Another patient with non-SCLC-pLEMS lacked an exact tumor diagnosis date and was therefore not shown in the figure. In two SCLC-pLEMS cases other malignancies were concurrent, one had epidermoid cancer of the lower lip diagnosed simultaneously with SCLC, while the other had cervical carcinoma 26 years before LEMS onset, which was considered unrelated

LEMS preceded tumor diagnosis in 33% of SCLC-pLEMS (median 2 months, range 1–25) and 30% of non-SCLC-pLEMS (median 15 months, range 1–51), was concurrent in 25% and 30%, and followed tumor diagnosis in the remainder. Median time from tumor to LEMS onset was 1 month (range 1–5) in SCLC-pLEMS and 10 months (range 1–21) in non-SCLC-pLEMS (Fig. 2).

Age at LEMS onset was significantly higher in SCLC-pLEMS (median 64 years, range 50–75) compared to aiLEMS (median 56 years, range 13–86; *p* = 0.029), while no such difference was found compared to non-SCLC-pLEMS (median 59 years, range 34–81). A male predominance was observed in non-SCLC-pLEMS (73%) compared to SCLC-pLEMS (42%) and aiLEMS (50%) (Supplemental Fig. 1).

**Fig. 3:**
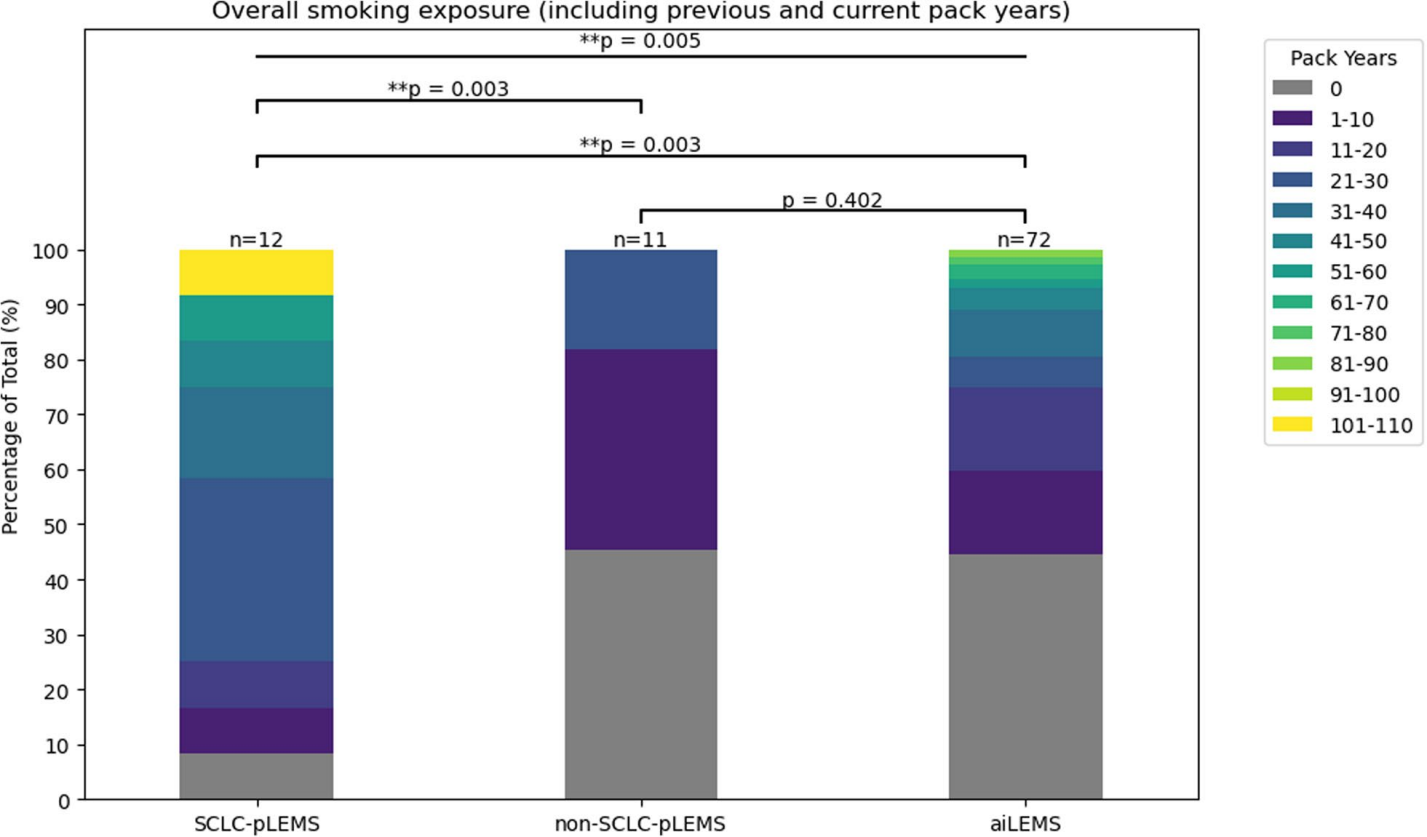
Stacked bar plot displaying previous smoking behavior, expressed in pack-years (PY), for patients with SCLC-pLEMS, non-SCLC-pLEMS, and aiLEMS enrolled in the European LEMS registry. Patients were grouped according to total PY, and values were normalized to percentage within each group. Group sizes (n) are indicated above each bar. Kruskal–Wallis test and Mann–Whitney U test were used as appropriate. *p ≤ 0.05,^**^p ≤ 0.01

There were no significant differences in clinical characteristics (muscle strength, reflexes, bulbar symptoms, ANS function) between the groups (Table 1). A history of smoking and higher numbers of pack-years (PY) were significantly more frequent in patients with SCLC-pLEMS (92%, median 30 PY, range 8–108) compared to non-SCLC-pLEMS (55%, median 6 PY, range 4–30, *p* = 0.003) and aiLEMS (56%, median 20 PY, range 4–82, *p* = 0.003) (Fig. 3).

**Table 1:** Baseline characteristics of patients in the European LEMS registry

Number of patients	Total	SCLC-pLEMS	non-SCLC-pLEMS	aiLEMS
	95	12	11	72
**Demographics**				
Age at onset	57 [13–86]	64 [50–75]	59 [34–81]	56 [13–86]
Age at enrollment	62 [21–90]	64 [52–76]	62 [38–84]	61 [21–90]
Male sex	49 (54)	5/12 (42)	9/11 (73)	36/72 (50)
**Clinical pattern**				
Muscle weakness ever^a)^	69/70 (99)	9/9 (100)	9/9 (100)	51/52 (98)
Classical pattern of muscle weakness^b)^	57/68 (84)	6/9 (67)	7/9 (78)	44/50 (88)
Reduced reflexes in UE ever	50/72 (69)	4/9 (44)	10/11 (91)	36/52 (63)
Reduced reflexes in LE ever	54/70 (77)	5/9 (56)	11/11 (100)	38/50 (76)
Bulbar weakness ever^c)^	29/76 (38)	3/7 (43)	2/6 (33)	24/63 (38)
ANS dysfunction ever^d)^	63/68 (93)	6/6 (100)	7/8 (88)	50/54 (92)
Further PNS	2/95 (2)	0/0 (0)	2/11 (18)	-
**Electrophysiology data**	39 (41)	4/12 (33)	5/11 (45)	29/72 (40)
CMAP amplitude in mv	4 [0–18]	4 [0–14]	4 [1-5]	4 [0–18]
Decrement positive	18/23 (78)	2/3 (67)	2/2 (100)	14/18 (78)
Decrement %	18 [1–52]	15 [6-24]	21 [2–52]	20 [0–51]
Increment positive	18/20 (90)	3/3 (100)	2/2 (100)	13/16 (81)
Increment %	115 [0–325]	114 [20–325]	102 [28–322]	79 [0–90]
**Antibody diagnostic**				
P/Q VGCC Abs positive	27/27	2/2 (100)	2/2 (100)	20/23 (87)
** LEMS specific treatment**^f)^				
3,4-DAP/ 3,4- DAPP	89/94 (95)	11/11 (100)	10/11 (91)	68/72 (94)
4-Aminopyridine (4-AP)	1/94 (1)	1/11 (9)	0/11 (0)	0/72 (0)
Cholinesterase inhibitor	16/94 (17)	5/11 (45)	10/11 (91)	36/72 (50)
Steroids	44/94 (47)	2/11 (18)	4/11 (36)	38/72 (53)
Non-steroidal IST	35/94 (37)	0/11 (0)	3/11 (27)	32/72 (44)
Rituximab Cyclophosphamide	10/94 (11) 1/94 (1)	0/11 (0) 0/11 (0)	3/11 (27) 0/11 (0)	7/72 (10) 1/72 (1)
IVIG	28/94 (30)	2/11 (18)	5/11 (45)	21/72 (29)
Plasma exchange	8/83 (8)	1/11 (9)	2/11 (18)	5/72 (7)
**Tumor therapy**				
Any Tumor therapy	22/23 (96)	12/12 (100)	5/9 (56)	–
Chemotherapy	15/23 (65)	12/12 (100)	3/9 (33)	–
Radiotherapy	7/23	6/12 (50)	3/9 (33)	–
Surgery	2/23	1/12 (8)	4/9 (44)	–

Data are presented as median [range] or frequency (%) as appropriate. No significant differences were found between patients with SCLC-pLEMS, non-SCLC-pLEMS and aiLEMS (Chi square test, Kruskal Wallis test)

^a)^Muscle strength was measured by medical research counsil (MRC) across 12 muscle groups

^b)^Defined as predominant proximal leg weakness exceeding arm or bulbar involvement

^c)^Present if at least one of the following positive: Dysarthria, dysphagia, chewing, neck weakness

^d)^Present if at least one of the following positive: dry eyes, dry mouth, pupil reflex abnormal, constipation, bladder dysfunction, orthostatic intolerance

^e)^Diagnostic criteria included: (1) clinical presentation with proximal leg-predominant weakness, (2) characteristic electrophysiological findings (decrement/increment), and (3) presence of P/Q-type VGCC antibodies at time of study inclusion

^e)^LEMS specific treatments were either concurrently or sequentially given over study period

*ANS* = autonomic nervous system, *BL* = baseline, *CMAP* = compound muscle ation potential, *IVIG* = intravenous immunoglobulin, *LE* = lower extremities, *LEMS* = Lambert-Eaton myasthenic syndrome, *IST* = immunosuppressive treatment, *MRC* = Medical Research Council, *y* = years; *3,4-DAP *= 3,4-Diaminopyridine, *3,4-DAPP* = 3,4 Diaminopyridine phosphate, *PNS* = paraneoplastic neurologic syndrome, *UE* = upper extremities

P/Q-type VGCC Ab testing at study enrollement was done in 27 patients (aiLEMS n = 23, median titer 146 pmol/L, range 3–1500; SCLC-pLEMS n = 2, titers 320 and 452 pmol/L; non-SCLC-pLEMS n = 2, titers 75 and 44 pmol/L). In the overall LEMS cohort, median compound muscle action potential (CMAP) was 3.9 mV (range 0–18), median decrement was 18% (range 1–51; normal < 8%[27]), and median increment was 115% (range 0–325; normal < 60% [27]), with comparable values among patients with available data in the aiLEMS (n = 29), non-SCLC-LEMS (n = 5), SCLC-pLEMS (n = 4) groups (Table 1).

**Fig. 4:**
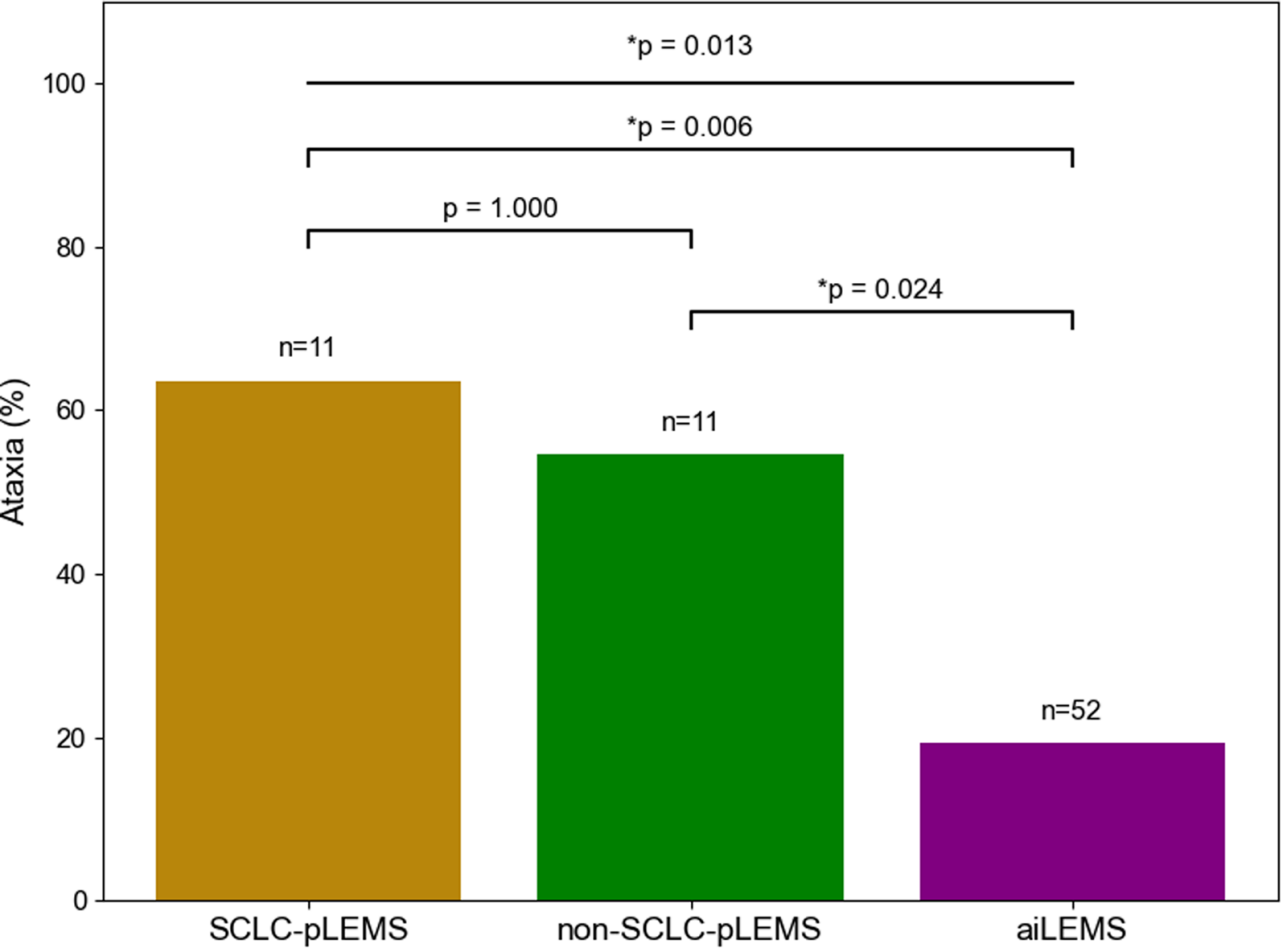
Bar graph of ataxia at any time during study in patients with SCLC-pLEMS, non-SCLC-pLEMS, and aiLEMS. Chi-square test and Fisher’s exact test were used as appropriate. ^*^p ≤ 0.05,,^**^p ≤ 0.01

Ataxia at any time during study participation was significantly more frequent in SCLC-pLEMS (7/11, 64%) and non-SCLC-pLEMS (6/11, 55%) than in aiLEMS (10/52, 19%, *p* = 0.006 and *p* = 0.024, Fig. 4). Additional paraneoplastic syndromes included rapid progressive cerebellar syndrome (RPCS) in 1 of 2 MCC patients, as well as neuropathy and myasthenia gravis associated with anti-CV2-antibodies in a patient with malignant thymoma.

Amifampridine or Amifampridine-Phosphate were taken in most patients (89 of 94, 95%). Cholinesterase inhibitors were more frequently given in non-SCLC-pLEMS (91%) than in aiLEMS (50%) and SCLC-pLEMS (45%). Steroids and non-steroidal immunosuppressants were most often used in aiLEMS (53% and 44%), less so in non-SCLC-pLEMS (46% and 27%) and SCLC-pLEMS (18% and 0%). IVIG was most frequently administered in non-SCLC-pLEMS (54%), compared to aiLEMS (29%) and SCLC-pLEMS (18%).

**Fig. 5:**
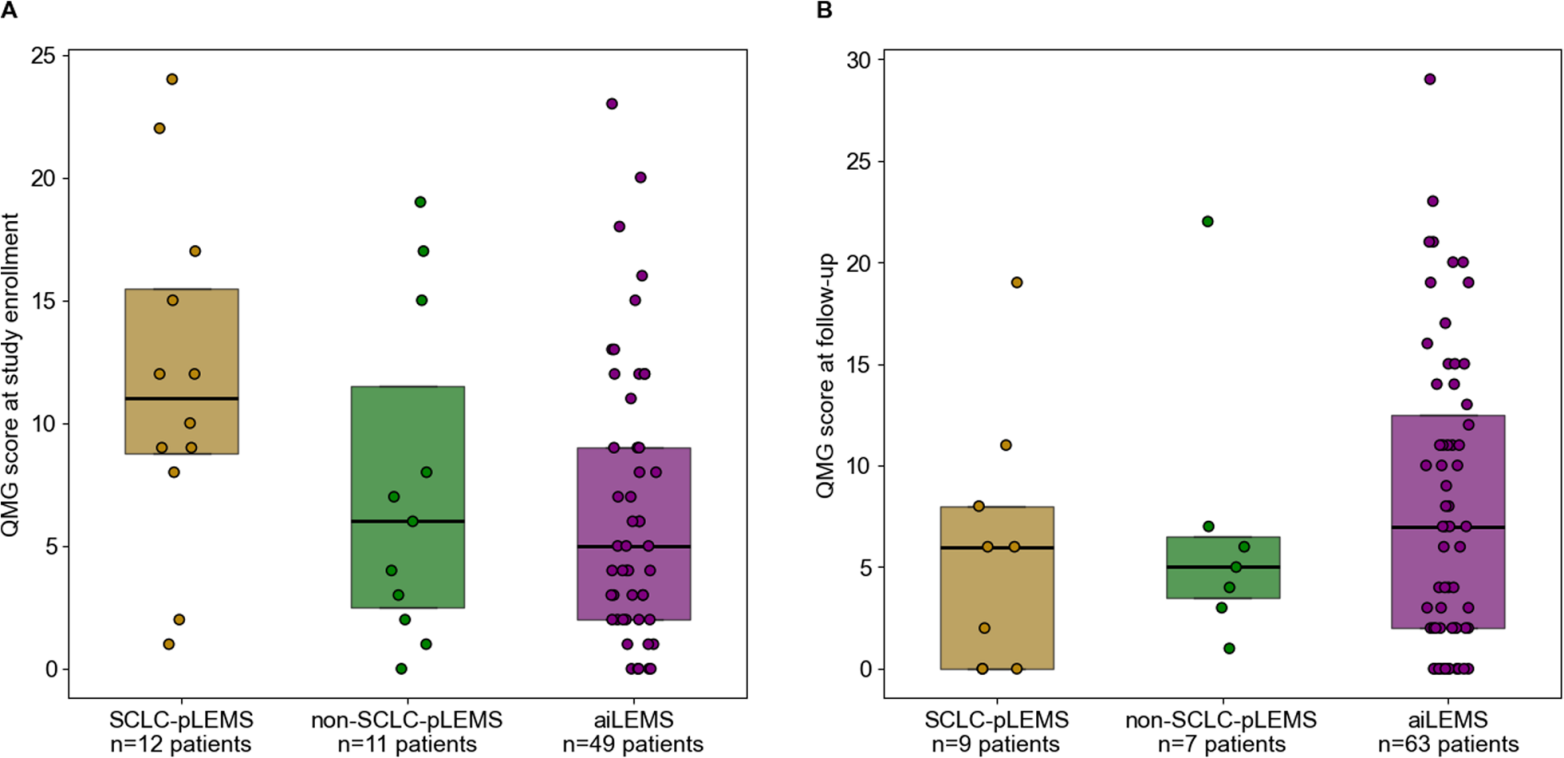
QMG scores at study enrollement (**A**) and last follow-up (**B**)

At study enrollment, SCLC-pLEMS had the highest QMG scores (median 12, range 1–24), compared to non-SCLC-pLEMS (median 6, range 0–19) and aiLEMS (median 5, range 0–23, Fig. 5A). In most SCLC-pLEMS patients (n = 10), tumor therapy had only recently been initiated (median 3 months, range 1–6), which likely contributed to the higher scores. Only two SCLC-pLEMS patients who had completed tumor therapy 2 and 4 years before study inclusion showed markedly lower QMG scores (1 and 2), indicating clinical improvement, although initial QMG values at disease onset were not available. Likewise, in non-SCLC-pLEMS, 3 with high QMG scores (15 -19) just begun tumor therapy, while 3 patients (QMG 6–8) had already undergone longer treatment (median 12 months, range 6–12), and five had completed therapy before enrollment, probably explaining lower QMG scores (0–4).

Individual courses in non-SCLC-pLEMS, fluctuated with tumor activitiy: two patients developed tumor recurrence during the study with a substantial worsening of LEMS (QMG increasing from 4 to 19 and from 11 to 26), both improving after reinitiation of tumor treatment (to 7 and 1, respectively). In one patient, QMG improved from 8 to 3 after tumor treatment had been completed during study follow-up, while another developed a new tumor with a rise in QMG from 0 to 5 that improved after treatment to 1. At last follow-up- median 44 months (range 4–105) for SCLC-pLEMS, 23 months (range 2–56) for non-SCLC-pLEMS, and 35 months (range 1–102) for aiLEMS)—all patients with pLEMS had completed tumor therapy, and QMG scores were largely comparable across subtypes (median 6 (range 0–19 in SCLC-pLEMS, 5 (range 1–22) in non-SCLC-pLEMS, and 7 (range 0–29) in aiLEMS, Fig. 5B).

In line with lower QMG scores, functional limitations in daily activities at study enrollment were less frequently reported in non-SCLC-pLEMS, with 40–50% per task, compared to 67–100% in SCLC-pLEMS and 49–82% in aiLEMS. (Supplement Table 1).

Overall, 35 of 95 patients (37%) discontinued treatment prematurely, with 15 patients (16%) dying during follow-up (SCLC-pLEMS n = 7, non-SCLC-pLEMS n = 6, aiLEMS n = 2). (Table 1). All causes of death in SCLC-pLEMS patients were tumor-related, whereas in most non-SCLC-pLEMS (5/6) and both aiLEMS cases, non-malignancy-related causes such as cardiac arrest (n = 3), severe infection (n = 3), and renal failure (n = 1) were reported. Survival tended to be lower in SCLC-pLEMS (median 24 months, range 9–155) compared to non-SCLC-pLEMS (median 75 months, range 1–145). Both pLEMS groups showed significantly reduced survival compared to aiLEMS (*p* < 0.001), for which, median survival could not be estimated due to the low number of deaths during the follow-up period (Supplemental Fig. 2).

### Systematic review of the literature

A total of 767 papers were screened, identifying 91 papers describing 126 LEMS patients with 137 tumors reported between 1979 and 2024 (Fig. 1, Supplemental Table 2). The most frequent were non-small cell lung cancer (NSCLC) (25/137, 18%), MCC (18/137, 13%), lymphoproliferative disorders (15/137, 11%), and both prostate cancer and thymoma (10/137 each, 8%).

**Table 2:** Clinical, laboratory, treatment, and outcome profiles of non-SCLC-pLEMS patients and medical history available identified through the systematic literature review

*N°*	*82*
Male Age (y)	50/79 (63) 62 [2–78]
Smoking before cancer	23/37 (62)
**Clinical symptoms**	
Muscle weakness	73/76 (96)
Fatigue	27/76 (36)
Reduced reflexes	45/76 (59)
Autonomic dysfunction	46/76 (61)
Muscle pain	7/76 (9)
Ocular symptoms	34/76 (45)
Bulbar symptoms	30/76 (39)
Weight lost	18/76 (24)
Ataxia	21/76 (28)
**Further paraneoplastic syndrome**	15/79 (19)
Cerebellar syndrome	4/79 (5)
Myasthenia gravis	4/79 (5)
Neuropathy	4/79 (5)
Scleroderma	1/79 (1)
Dermatomyositis	1/79 (1)
Limbic encephalitis	1/79 (1)
**LEMS Diagnostic assessments**	
Electrophysiology positive	68/71 (96)
P/Q-VGCC P/Q Ab positive	43/51 (84)
P/Q-VGCC Abs titer (median in pmol/L [range])	24/51 (47), 177 [32–23000]
**Tumor screening**	
Chest CT	72/72 (100)
FDG-PET/CT	18/68 (26)
**LEMS treatmen**t ^a)^	
Symptomatic treatment	38/75 (51)
3,4-DAP/3,4-DAPP	21/75 (28)
4-Aminopyridine	4/75 (5)
Cholinesterase inhibitor	26/75 (35)
Steroids	24/75 (32)
Non-steroidal IST	9/75 (12)
IVIG	16/75 (21)
Plasma exchange	10/75 (13)
Rituximab	3/75 (4)
Cyclophosphamide	2/75 (3)
**Tumor therapy** ^a)^	
Any tumor therapy	68/71 (96)
Surgery	50/71 (70)
Chemotherapy	34/71 (48)
Radiotherapy	31/71 (43)
Immune checkpoint inhibitor	5/71 (7)
**Outcome after tumor treatment**	
Full recovery	23/52 (44)
Partial recovery	23/52 (44)
No change	5/52 (10)
Deterioration	1/52 (2)
Died due to tumor progression	13/82 (16)

Data are presented as median [range] or frequency (%) as appropriate. Abbreviations: *CT* = computer tomography, *IVIG* = intravenous immunoglobulin, *IST* = immunosuppressive treatment, *m *= months, *y* = years

^a)^Patient can be in more than one group

Of these, 115 patients were extracted as literature-reported cases, while 11 cases originated from our European LEMS registry [28], which have not been previously described as a separate non-SCLC-pLEMS group that and were therefore excluded from the literature cohort. Among the 115 literature cases, patient-level data were available for 82, although the level of detail varied considerably (Table 2). Median age was 62 years (range 2–78), and 62% were male (Fig. 3). Electrophysiological data (n = 71) showed abnormalities (decrement of > 10% and/or a marked increment relative to rest) in 96%. P/Q-type VGCC Abs (n = 51) were positive in 84%, with titers available in 24 patients (median 177 pmol/L (range 32–23.000, normal range < 20 pmol/L[30]). An 18-F fluorodeoxyglucose positron emission tomography (FDG-PET) was used in 18/68 (26%) patients with tumor screening data. Among 81 non-SCLC-pLEMS patients with available biopsy data, 40 patients (49%) had a neuroendocrine neoplasm. Notably, radid progressive cerebellar syndrome (RPCS) was reported in 25% of MCC patients (4/16), while further PNS included myasthenia gravis (n = 4), sensory neuropathy (n = 4), scleroderma (n = 1), dermatomyositis (n = 1), Table 2 and limbic encephalitis (n = 1).

Among those with available data (n = 62), LEMS preceded tumor diagnosis in 35% (median 6 months, range 1–72), was concurrent in 33%, and followed tumor diagnosis in 32% (median 11 months, range 1–60, Fig. 2). Treatment data was available for 75 literature-reported non-SCLC-pLEMS patients. Symptomatic therapy was given in 28% (amifampridine) and 35% (cholinesterase inhibitors), steroids in 32%, non-steroidal immunosuppressants in 12%, exacerbation therapies with IVIG in 21%, while tumor-directed therapy was administered in 91%. Outcome data (n = 52) showed clinical improvement in 88% after tumor treatment—half with full, half with partial recovery of LEMS symptoms (Table 2). Among these patients, the most common tumor types were MCC (13/52, 25%), NSCLC (10/52, 19%), and thymoma (7/52, 13%). Subgroup analysis found no significant differences between neuroendocrine and non-neuroendocrine non-SCLC-pLEMS regarding clinical presentation, treatment, and outcomes (Supplemental Table 3).

Apart from more frequent ataxia in registry patients (55% vs. 28%), no substantial differences in demographics (Supplemental Fig. 1), smoking behavior, time relationship between LEMS onset and tumor diagnosis, or other clinical features were observed, suggesting that literature-reported non-SCLC-pLEMS cases are representative of our registry cohort. Compared to registry non-SCLC-pLEMS patients, symptomatic and immunosuppressive therapies appeared less frequently used in the literature cohort, whereas tumor-directed treatments were similarly common.

## Discussion

This study comprehensively investigated the clinical phenotype of non-SCLC-pLEMS, in comparison to SCLC-pLEMS and aiLEMS. The temporal relationship of LEMS diagnosis before or after tumor detection was similar across SCLC-pLEMS, and non-SCLC-pLEMS, with longer intervals tending to occur in non-SCLC-pLEMS in both registry and literature. Ataxia was more common in SCLC-pLEMS and non-SCLC-pLEMS, compared to aiLEMS, while QMG scores in both pLEMS groups largely reflected tumor treatment status, improving or stabilizing with therapy and worsening with recurrence or progression. Consistently, most of literature-reported non-SCLC-pLEMS patients with available data improved after tumor therapy, supporting a paraneoplastic origin. However, for several non-SCLC tumors, scarce outcome data and LEMS symptoms response to tumor treatment was not documented, some associations may be coincidental, with neurologic symptoms misattributed to tumor progression rather than LEMS.

This study adds to current understanding of non-SCLC malignancies associated with LEMS. In our registry, intervals from LEMS onset to cancer diagnosis were − 25 to + 5 months (SCLC-pLEMS) and − 51 to + 21 months (non-SCLC-pLEMS), similar to our literature cohort of patients with non-SCLC-pLEMS (− 6 to + 11 months). Unlike a prior pooled analysis of all pLEMS showing 86% diagnosed before cancer [31], only ~ one-third of SCLC-pLEMS and non-SCLC-pLEMS in our registry and literature cohorts preceded cancer detection. These differences may reflect variations in study design (systematic review versus registry data), differences in tumor biology, or variability in tumor screening strategies. When considering the temporal association within ± 12 months of LEMS onset, the proportion of tumors detected in this 2-year time frame was 91% (9/11) in SCLC-pLEMS, 40% (4/10) in the non-SCLC-pLEMS registry cohort, and 86% (55/64) in the non-SCLC-pLEMS literature cohort. Among non-SCLC tumor entities (registry and literature) with sufficient numbers and available data, this close temporal relationship was most prominent for MCC (73%, 11/15), thymoma (100%, 9/9), NSCLC (85%, 11/13), lymphoproliferative disorders (86%, 6/7) and prostate cancer (75%, 3/4), but less consistent for colon (50%, 2/4) and breast cancer (20%, 1/5). These findings suggest that certain tumor types—particularly MCC, thymoma, NSCLC, lymphoproliferative disorders and prostate cancer—show a strong temporal relationship with LEMS onset, supporting a paraneoplastic link, whereas associations with other malignancies may be coincidental.

At enrollment, QMG reflected treatment status rather than differences between SCLC-pLEMS and non-SCLC-pLEMS—being higher shortly after treatment initiation and lower with prolonged or completed therapy. On follow-up, symptoms improved/stabilized with tumor control and worsened with recurrence. Consistently, literature data, showed that 88% of non-SCLC-pLEMS patients with available data showed partial or full recovery of LEMS after tumor therapy, strongly supporting a paraneoplastic origin.

Expanding awareness of LEMS in non-SCLC tumors, particularly extrapulmonary neuroendocrine malignancies, such as MCC, has grown in recent years [19, 32]. Neuroendocrine non-SCLC carcinomas were present in various organs (including prostate, cervix, pancreas, and skin), accounting for 49% of all non-SCLC-pLEMS cases with available histopathology. Consistent with this broader spectrum, a Turkish study reported that only about half of pLEMS cases were linked to SCLC, with NSCLC being the most frequent non-SCLC malignancy [26].

In SCLC-pLEMS, a causal link is supported by the fact that P/Q VGCC antigen is functionally expressed in the surface membrane of these tumor cells [33], and Abs derived from LEMS patients can dose-dependently block VGCC activity on SCLC cell surfaces [34]. Although VGCC expression within non-SCLC tumors has only been reported in isolated LEMS cases, recent transcriptomic and immunohistochemical data suggest that several VGCC subtypes, including P/Q-type channels, are indeed expressed in multiple tumors such as prostate and breast cancer [35–37]. These findings support further investigation of VGCC expression in non-SCLC tumors and may ultimately inform reclassification of non-SCLC-pLEMS beyond “*possible* PNS” according to the current Graus criteria [38].

Registry data showed that ataxia was more frequent in SCLC-pLEMS and non-SCLC-pLEMS compared to aiLEMS, consistent with previous reports indicating a higher prevalence of cerebellar ataxia in SCLC-pLEMS than in aiLEMS [29, 39]. P/Q-type VGCC Abs, highly expressed in cerebellar Purkinje and granule cells, have been implicated in cerebellar degeneration and should be considered in the context of paraneoplastic cerebellar degeneration [40, 41]. Beyond SCLC, our data suggest that certain tumor types, particularly MCC, may predispose LEMS patients to additional paraneoplastic cerebellar syndrome. Likewise, MCC shares clinical and immunologic profiles with SCLC, including a neuroendocrine origin and expression of antigens, which may underlie similar paraneoplastic manifestations [19, 42–44]. These findings highlight the need for prospective studies to determine whether ataxia in pLEMS is part of the LEMS spectrum or a distinct paraneoplastic syndrome. Despite the high prevalence of P/Q-type VGCC Abs, ataxia appears limited to a subset of pLEMS patients and is rare in aiLEMS, as shown in our registry and prior studies [45, 46]. Notably, in our registry, none of the aiLEMS patients with ataxia developed a tumor during 35 months of follow-up, arguing against an increased cancer risk associated with ataxia in aiLEMS patients.

While the Delta-P-Score helps predict SCLC probability and guides malignancy screening in LEMS, it is specific to lung cancers [47]. Due to our study's retrospective design and limited sample size, we could not validate it for non-SCLC-pLEMS. This points to developing comparable models for non-SCLC malignancies, perhaps incorporating ataxia to help distinguish pLEMS from aiLEMS. Besides, the broad spectrum of tumor types associated with LEMS poses a diagnostic challenge, as screening strategies often depend on the specific malignancy [48]. In patients with unremarkable (thoracic) CT findings, some experts recommend performing an FDG-PET as part of the initial screening for malignancy [5, 48]. In our systematic review, only 26% of non-SCLC-pLEMS patients underwent FDG-PET, suggesting that a lower threshold for PET screening could have facilitated earlier tumor detection in more cases.

Our registry data revealed that steroidal and non-steroidal immunosuppressive treatments were more common in aiLEMS than in SCLC-pLEMS, whereas immunomodulatory therapies (IVIG, plasma exchange), and rituximab were more often administered in non-SCLC-pLEMS. Our findings are in line with previous reports showing azathioprine-steroid combinations in 70% of aiLEMS versus 44% of SCLC-pLEMS cases [49]. These results suggest that tailored therapeutic strategies may be needed for different LEMS subgroups, depending on tumor types. However small sample sizes prevented significant conclusions.

This study has limitations. In our systematic review, non-SCLC-pLEMS cases were collected over 40 years, while registry data reflect more recent diagnostic and treatment advances. The registry includes only a subset of LEMS patients and is therefore incomplete and not fully representative. Aggressive malignancies like SCLC may be underrepresented, as patients often proceed directly to treatment, bypassing registry enrollment, which may bias tumor frequency estimates. Accordingly, the proportion of tumor-associated LEMS in our registry appears lower than the ~ 60% pLEMS reported in earlier literature [6, 26], a difference that is likely influenced by registry-related selection and undercapture of rapidly treated cases such as SCLC. Besides, the broader tumor spectrum reported in non-SCLC-pLEMS may contribute to a numerically higher case count compared to SCLC-pLEMS, potentially leading to an overestimation of its relative prevalence, and small biopsies in NSCLC cases may have missed SCLC aspects, leading to misclassification. Small sample sizes in registry pLEMS limit statistical power and the generalizability. Importantly, registry data were based on re-assessments at the time of enrollment rather than on the original diagnostic work-up. Therefore, diagnostic criteria at disease onset could not be systematically evaluated across the three subgroups. Nonetheless, all patients fulfilled core LEMS criteria, including a compatible clinical presentation and at least one supportive finding—either electrophysiological evidence of a presynaptic disorder or P/Q-type VGCC antibodies. The lack of QMG data at disease onset restricted assessment of full tumor treatment response in pLEMS, however long-term follow-up still allowed evaluation of QMG changes in relation to therapy, relapse, and symptom course. While QMG was the only standardized quantitative outcome available, it is not optimal for detecting the characteristic lower-limb fatigability in LEMS. The triple timed up-and-go, now a well-established disease-specific functional score introduced later, better captures this feature [50] and should be considered in future prospective studies.

The assessment of ataxia, including the heel-to-knee test, may be limited in its interpretability in patients with muscular weakness. However, all neurological examinations were performed by experienced neurologists specialized in neuromuscular disorders, which helped to account for this potential confounder.

In the literature cohort, up to 50% of the data were missing, in 43%, the temporal association was uncertain and in 45% of cases outcome data after tumor therapy were missing, making a causal relationship with LEMS unclear in many cases.

## Conclusion

LEMS associated with non-SCLC tumors is rare and often underrecognized. Our data show that QMG scores in non-SCLC-pLEMS largely reflect tumor treatment status, improving or stabilizing after therapy and worsening at recurrence. Across registry and literature cohorts, most patients improved with tumor therapy, underscoring the clinical value of timely recognition. PET-CT should be considered when CT is negative to avoid missed tumor diagnoses, and ataxia may serve as red flag for pLEMS. Early diagnosis of all pLEMS is essential to ensure timely treatment and ultimately improve patient outcomes.

## Supplementary Information

Additional file 1.

## Data Availability

The data that support the findings of this study are available on request from the corresponding author. The data are not publicly available because of information that could compromise the privacy of research participants.

## Abbreviations

Ab: Antibody
aiLEMS: Autoimmune Lambert-Eaton myasthenic syndrome
ANS: Autonomic nervous system
CA: Cerebellar ataxia
CMAP: Compound muscle action potential
CT: Computer tomography
FDG-PET: 18-F fluorodeoxyglucose positron emission tomography
IA: Immunoadsorption
IVIG: Intravenous immunoglobulin
MCC: Merkel cell carcinoma;
NEN: Neuroendocrine neoplasms;
NSCLC: Non-small cell lung cancer
non-SCLC-pLEMS: Non small cell lung cancer associated paraneoplastic Lambert-Eaton myasthenic syndrome
PE: Plasma exchange
QMG-Score: Quantitative Myasthenia Gravis Score
RPCS: Rapid progressive cerebellar syndrome
pLEMS: Paraneoplastic Lambert Eaton myasthenic syndrome
PY: Pack-years
PRIMSA: Preferred Reporting Items for Systematic Reviews and Meta-Analyses
SCLC: Small cell lung cancer
SCLC-pLEMS: Small cell lung cancer associated paraneoplastic Lambert-Eaton myasthenic syndrome
VGCC: Voltage gated calcium channel
