# Supplementary material for: Paraneoplastic Lambert–Eaton myasthenic syndrome associated with non-small cell lung cancer: data from the European LEMS registry and systematic review
Source: Neurol Res Pract. 2025 Dec 8;7(1):95. doi: 10.1186/s42466-025-00453-5 (PMC12687528; doi:10.1186/s42466-025-00453-5)
Supplement: Supplementary file 1 — Additional file 1. [file 42466_2025_453_MOESM1_ESM.docx]

**Supplementary material**

**Tables**

**Supplement Table 1 Functional assessment outcomes at baseline for patients with non-SCLC-pLEMS, SCLC-pLEMS and aiLEMS enrolled in the European LEMS registry**

| **Limited/reduced function at baseline, n (%)** | **Total** | **SCLC-pLEMS** | **non-SCLC-pLEMS** | **aiLEMS** |
| --- | --- | --- | --- | --- |
| Walk upstairs | 46/57 (81) | 7/7 (100) | 2/5 (40) | 37/45 (82) |
| Cycle | 37/50 (74) | 6/6 (100) | 2/5 (40) | 29/39 (74) |
| Getting up from a low chair with arm support | 31/58 (53) | 7/8 (88) | 2/5 (40) | 22/45 (49) |
| Getting up from a low chair without arm support | 39/55 (71) | 5/6 (83) | 2/5 (40) | 32/44 (73) |
| Getting up from sitting on one knee | 38/50 (76) | 5/6 (83) | 2/4 (50) | 31/40 (78) |
| Getting up from squatting | 40/54 (74) | 5/6 (83) | 2/4 (50) | 33/44 (75) |
| Climb stairs with arm support | 33/57 (58) | 6/8 (75) | 2/5 (40) | 23/43 (54) |
| Climb stairs without arm support | 39/56 (70) | 6/7 (86) | 2/5 (40) | 31/44 (71) |
| Walking on toes | 30/55 (55) | 6/9 (67) | 2/5 (40) | 21/41 (51) |
| Walking on heels | 30/56 (54) | 6/9 (67) | 2/4 (50) | 22/43 (51) |
| Getting up from highchair with arm support | 29/55 (53) | 5/7 (71) | 4/5 (40) | 20/43 (47) |
| Getting up from a high chair without any arm support | 31/54 (57) | 5/6 (83) | 2/5 (40) | 24/43 (56) |

**Supplement Table 2 Cancers found in LEMS in registry and systematic literature search.**

|  | **Total** | **non-SCLC-pLEMS**  **in registry** | **non-SCLC-pLEMS in literature** |
| --- | --- | --- | --- |
| Number of all tumors ^a)^ | **137** | **13** | **124** |
| Cancer types  Non-small cell lung cancer  Merkel cell carcinoma  Lymphoproliferative disorders  Prostate cancer  Thymoma  Breast cancer  Colon cancer  Unknown primary origin  Mediastinal cancer  Ovarial cancer  Bladder cancer  Renal cancer  Pancreas cancer  Laryngeal carcinoma  Endometrial cancer  Leiomyosacroma (uterus)  Basalioma  Oropharyngeal cancer  Oesophagus cancer  Appendiceal carcinoma  Carcinoma of the seminal vesicles  Rectum cancer  Gall bladder cancer  Aggressive fibromatosis  Cervix carcinoma  Melanoma  Thyroid cancer  Tonsil cancer  Ameloblastoma  Pelvic tumor  Neuroblastoma  Kaposi sarcoma | 25  18  15  10  10  8  6  5  3  3  3  3  3  3  2  2  2  2  1  1  1  1  1  1  1  1  1  1  1  1  1  1 | 0  2  2  0  2  1  2  0  0  0  0  0  1  1  0  0  0  0  1  0  0  1  0  0  0  0  0  0  0  0  0  0 | 25  16  13  10  8  7  4  5  3  3  3  3  2  2  2  2  2  2  0  1  1  0  1  1  1  1  1  1  1  1  1  1 |
| **Geographic origin**  Europe  North America/Canada  Asia  Middle East  Australia/New Zealand | 63  42  24  6  2 | 12  0  0  0  0 | 51  42  24  6  2 |

1. In three patients two neoplasms occurred, in one patient two respectively three neoplasms

**Supplement Table 3 Clinical, laboratory, treatment, and outcome profiles of non-SCLC-pLEMS patients grouped for neuroendocrine and non-neuroendocrine tumor with patient-level data identified in systematic review**

|  | **Neuroendocrine tumor** | **Non-neuroendocrine tumor** |
| --- | --- | --- |
| N° | 40 | 41 |
| Male  Age (years) | 26/37 (70)  61 (3-78) | 22/40 (55)  64 (2-77) |
| Smoking before cancer | 13/19 (68) | 10/19 (53) |
| **Clinical symptoms**  Muscle weakness  Fatigue  Reduced reflexes  Autonomic dysfunction  Muscle pain  Ocular symptoms  Bulbar symptoms  Weight lost  Ataxia | 36/37 (97)  10/37 (27)  18/35 (51)  24/37 (65)  2/37 (5)  14/37 (38)  16/37 (43)  5/37 (14)  9/37 (24) | 36/38 (95)  18/38 (47)  26/35 (74)  21/38 (55)  5/38 (13)  19/38 (50)  14/38 (37)  13/38 (43)  12/38 (32) |
| **Further PNS**  RPCS  Myasthenia gravis  Neuropathy  Scleroderma  Dermatomyositis  Limbic encephalitis | 9/40 (23)  4/40 (10)  1/40 (3)  2/40 (6)  1/40 (3)  0 (0)  1/40 (3) | 6/41 (15)  0 (0)  3/41 (7)  2/41 (5)  0 (0)  1/41 (2)  0 (0) |
| **Diagnostic of LEMS**  Electrophysiology positive  P/Q VGCC Abs positive  P/Q-VGCC Abs titer (median in pmol/L [range]) | 36/36 (100)  23/25 (92)  13/25 (52), (120 [44-23.000] | 40/41 (98)  20/26 (77)  11/26 (42), (220 [32-777] |
| FDG/PET-CT | 11/37 (30) | 4/35 (11) |
| **LEMS treatment^a)^**  Symptomatic treatment  Steroids  Long-term IST  IVIG  Plasmapheresis  Rituximab  Cyclophosphamide | 15/36 (42)  6/36 (17)  4/36 (11)  10/36 (28)  6/36 (17)  2/36 (6)  2/36 (6) | 23/39 (59)  17/39 (44)  5/39 (13)  7/39 (18)  4/39 (10)  1/39 (3)  0 (0) |
| **Tumor therapy^a)^**  Surgery  Chemotherapy  Radiotherapy  ICI  Hormone therapy  No treatment | 25/39 (64)  23/39 (59)  23/39 (59)  5/39 (13)  2/39 (5)  0 (0) | 25/41 (61)  10/41 (24)  8/41 (20)  0 (0)  1/41 (2)  8/41 (20) |
| **Outcome after tumor treatment**  Full recovery  Partial recovery  No change  Deterioration | 15/33 (45)  14/33 (42)  3/33 (9)  1/33 (3) | 7/18 (39)  9/18 (50)  2/18 (11)  0 (0) |

Data are presented as median [range] or frequency (%) as appropriate. No significant differences were found between LEMS patients associated with neuroendocrine and non-neuroendocrine tumors (Chi square test, Mann Whitney U test).

Abbreviations: FDG PET = Fluorodeoxyglucose Positron Emission Tomography, ICI = immune checkpoint inhibitor; IVIG = intravenous immunoglobulin; Long-term IST = long-termin immunosuppressive treatment, RPCS = rapid progressive cerebellar syndrome

1. Patient can me in more than one group

**Figures**

**Supplemental Figure 1**


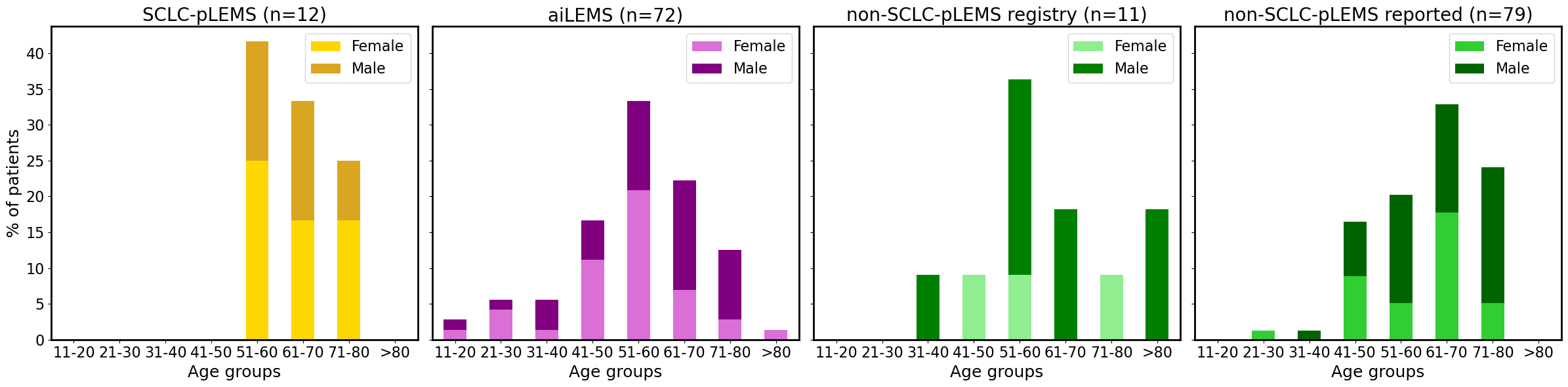


**Demographic profiles**

Stacked bar plots show the proportion of male and female patients across age groups for SCLC-pLEMS, aiLEMS and non-SCLC-pLEMS (registry-based and literature-reported). Tests applied: Chi-Square Test and Mann-Whitney U test

**Supplemental Figure 2**


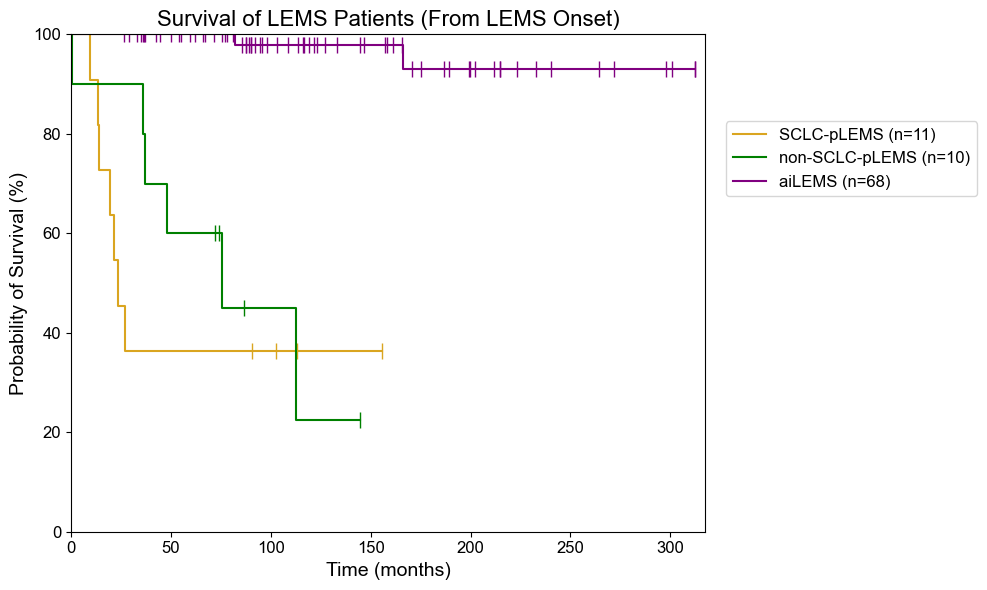


**Survival rate of patients with SCLC-pLEMS, non-SCLC-pLEMS, and aiLEMS in the European LEMS registry**

Kaplan-Meier curves show time from LEMS onset to death or last follow-up in patients with SCLC-pLEMS, non-SCLC-pLEMS, and aiLEMS.
